# Supplementary material for: EPHA4 signaling dysregulation links abnormal locomotion and the development of idiopathic scoliosis
Source: eLife. 2025 Jul 15;13:RP95324. doi: 10.7554/eLife.95324 (PMC12263152; doi:10.7554/eLife.95324)

**Original Western Blot Image** **of Three Replications for Figure 1F**

**1.**

pCDK5


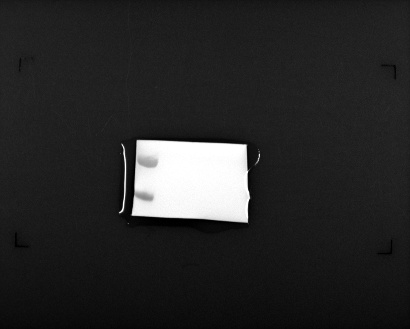

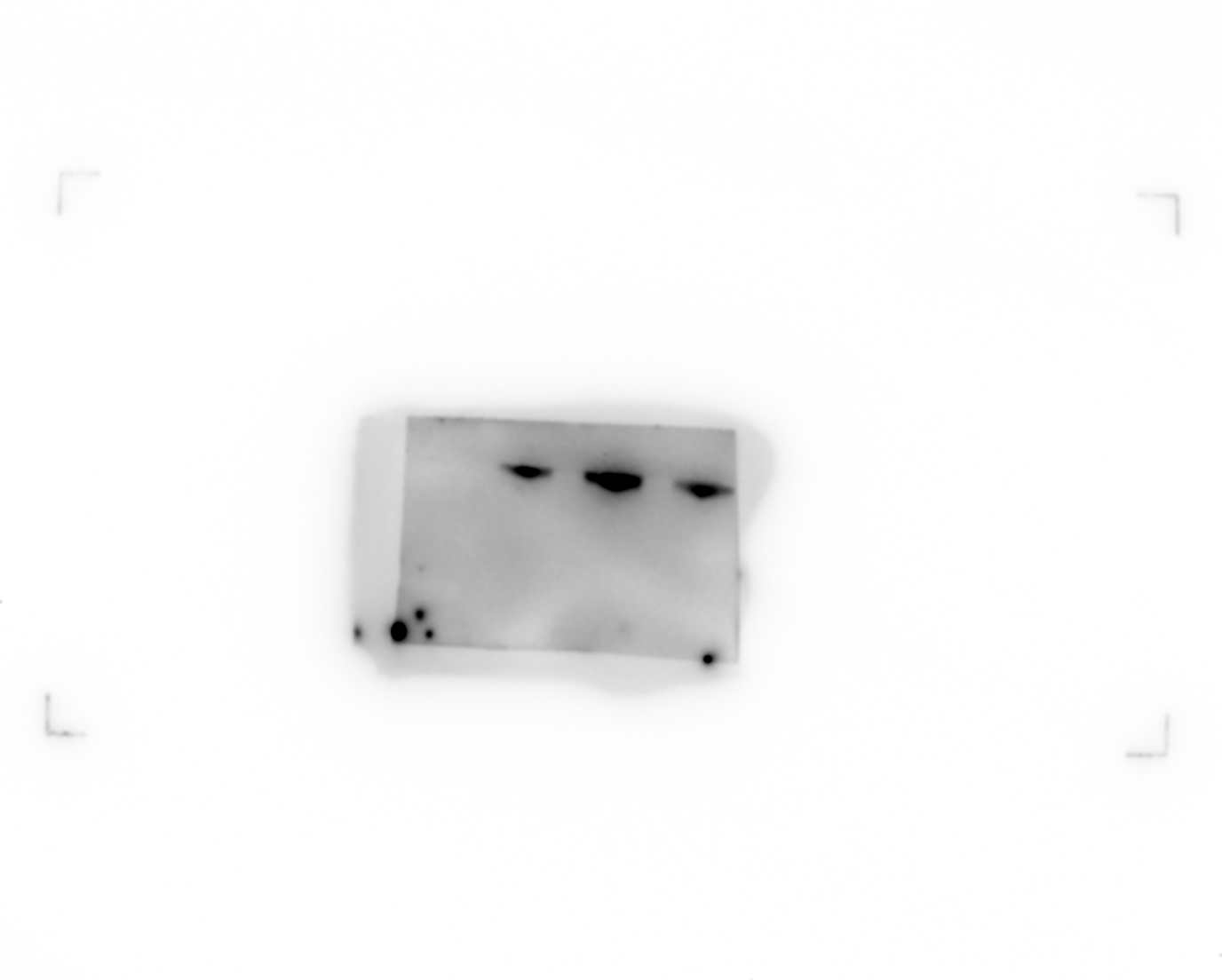


CDK5


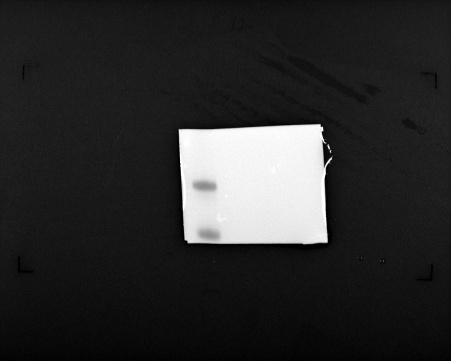

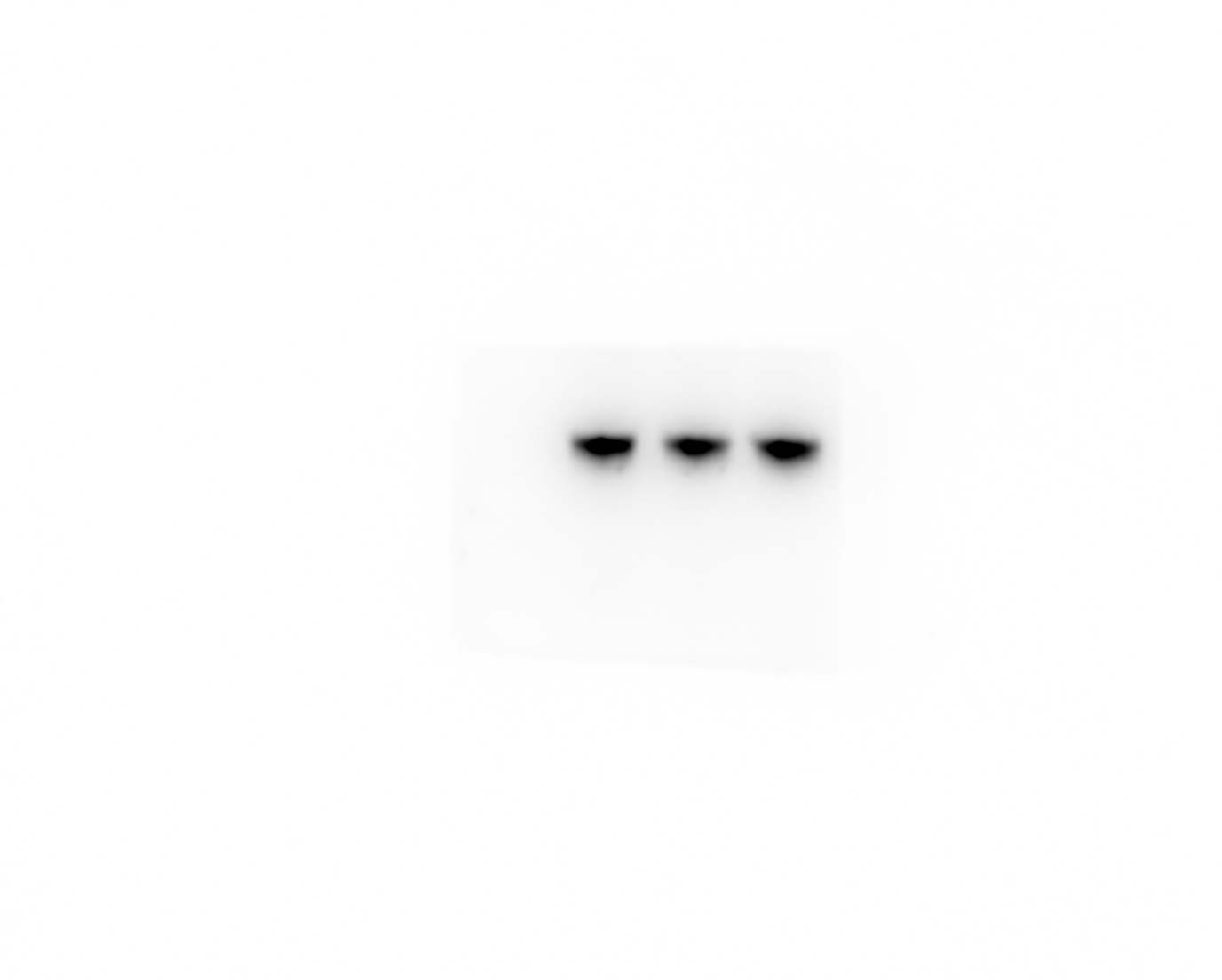


EPHA4


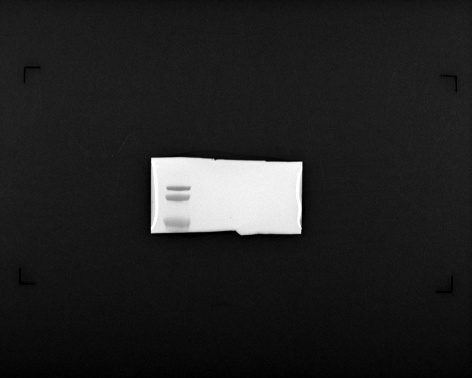

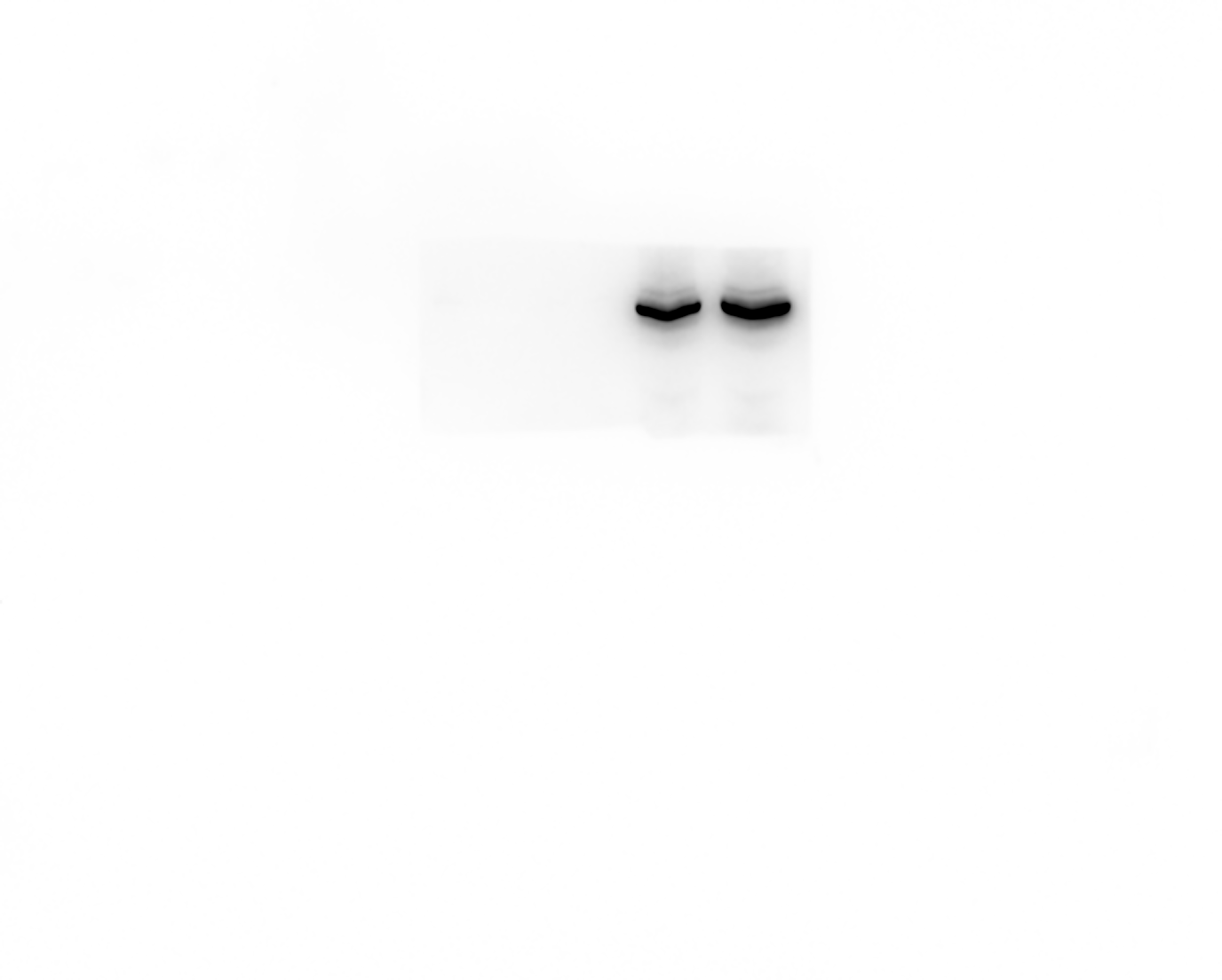


β-actin


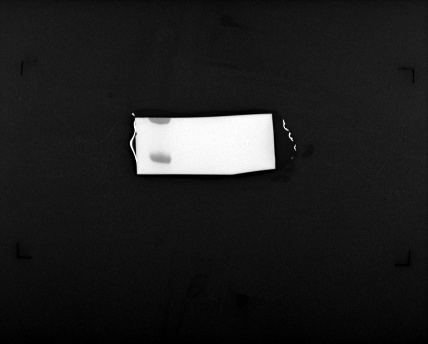

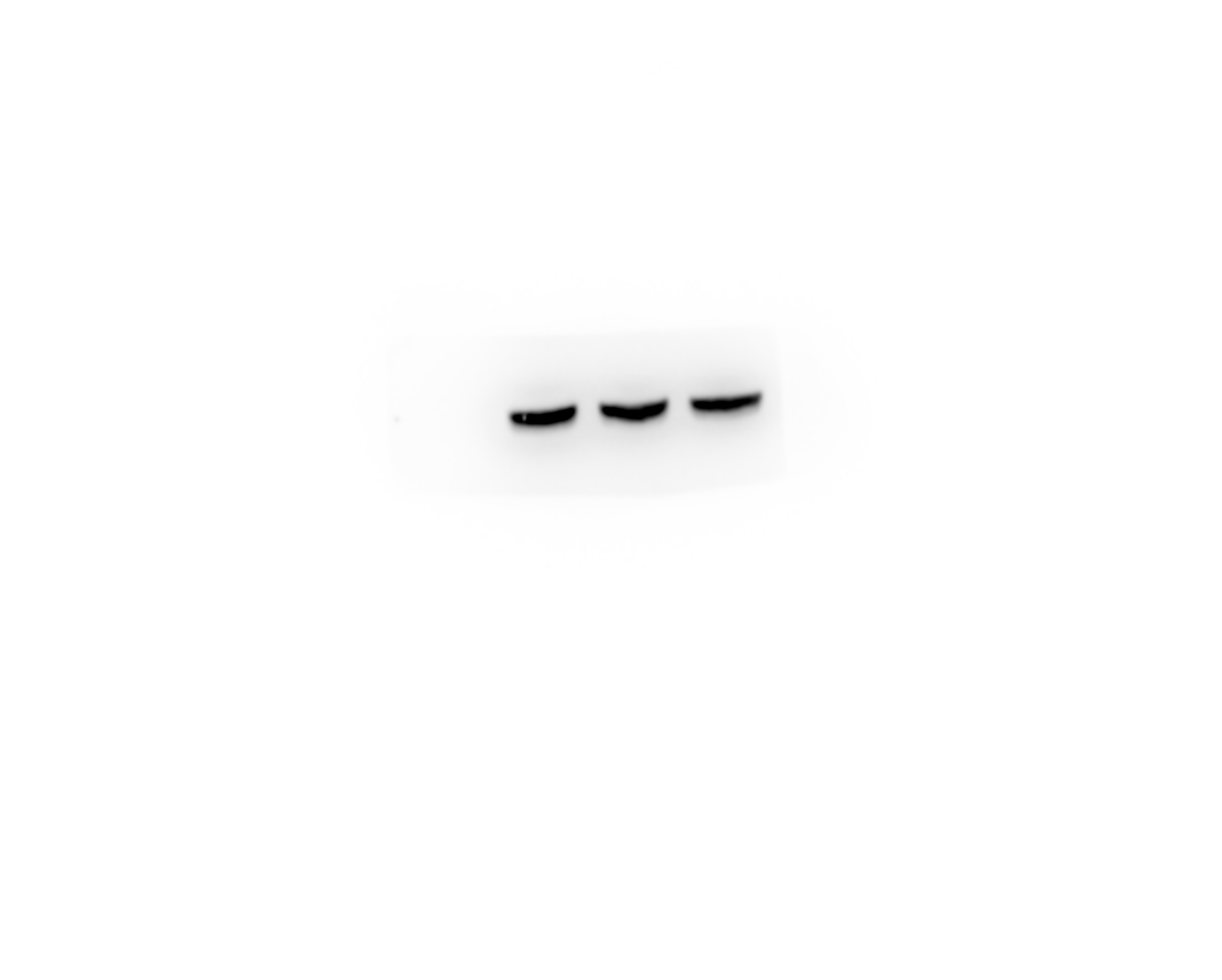


**2.**

pCDK5

###
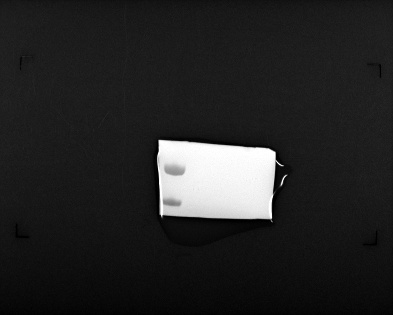

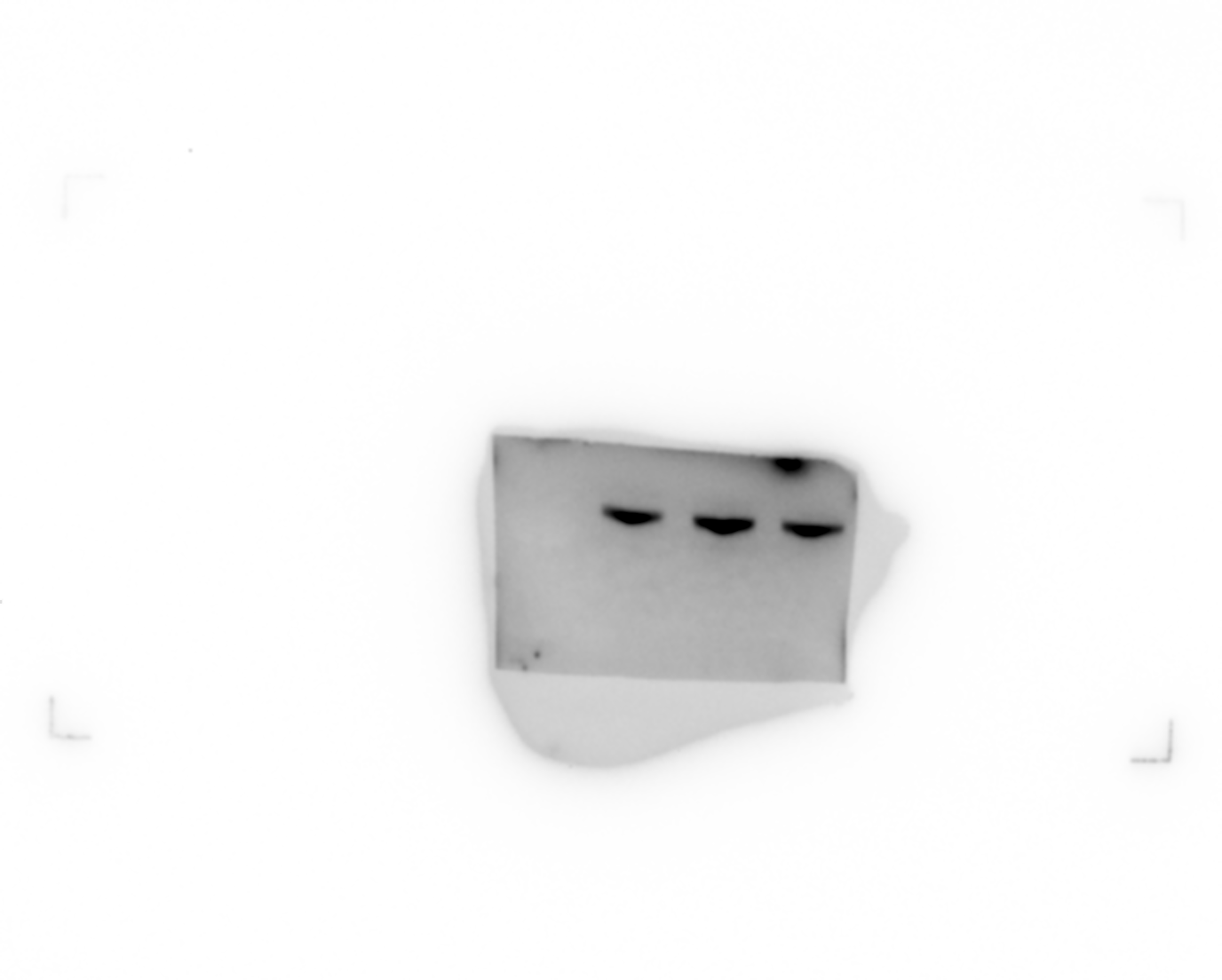


CDK5

###
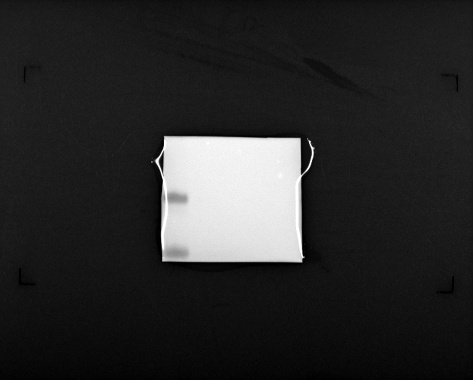

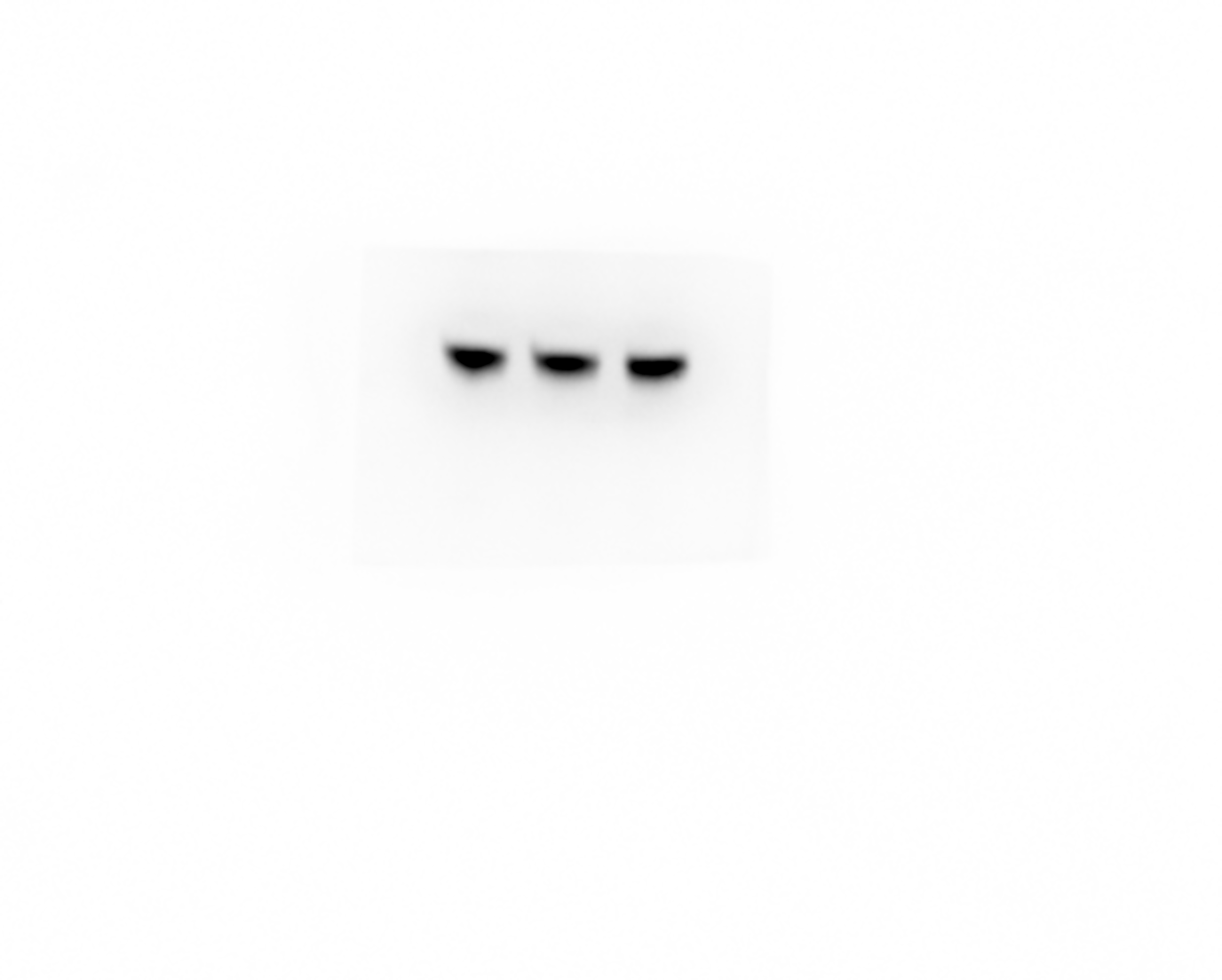


EPHA4

###
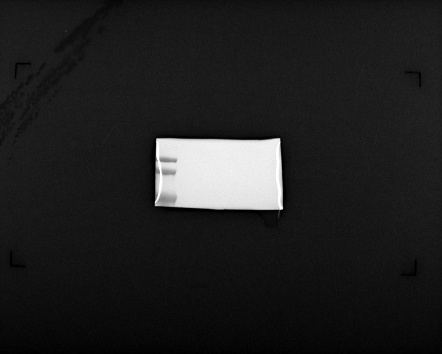

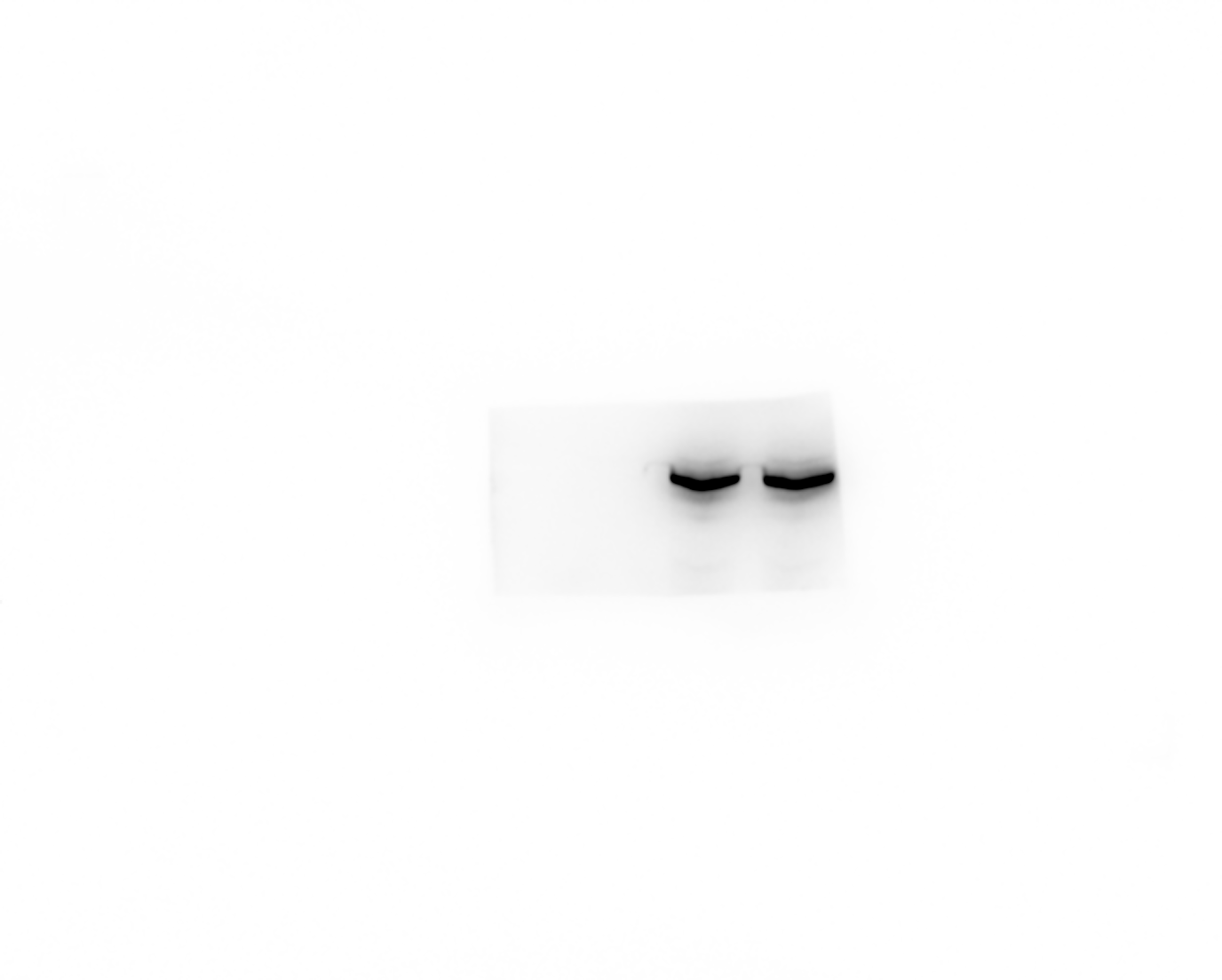


β-actin

###
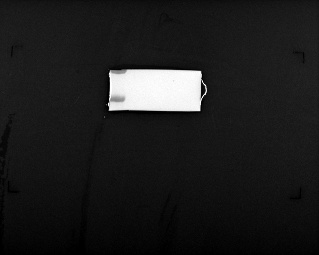

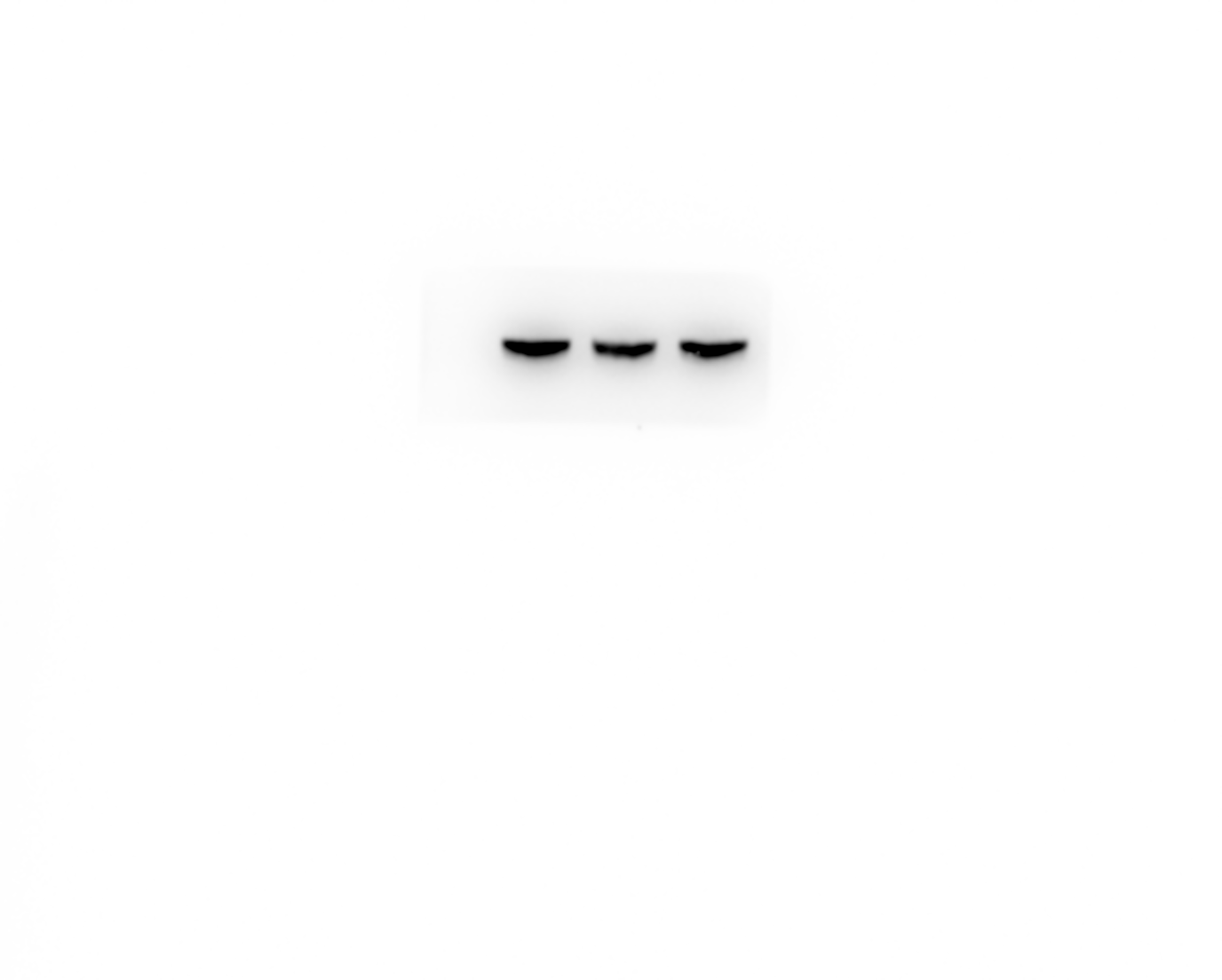


**3.**

pCDK5

###
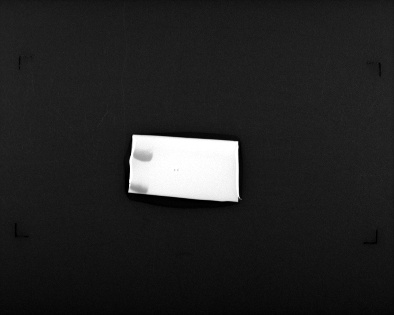

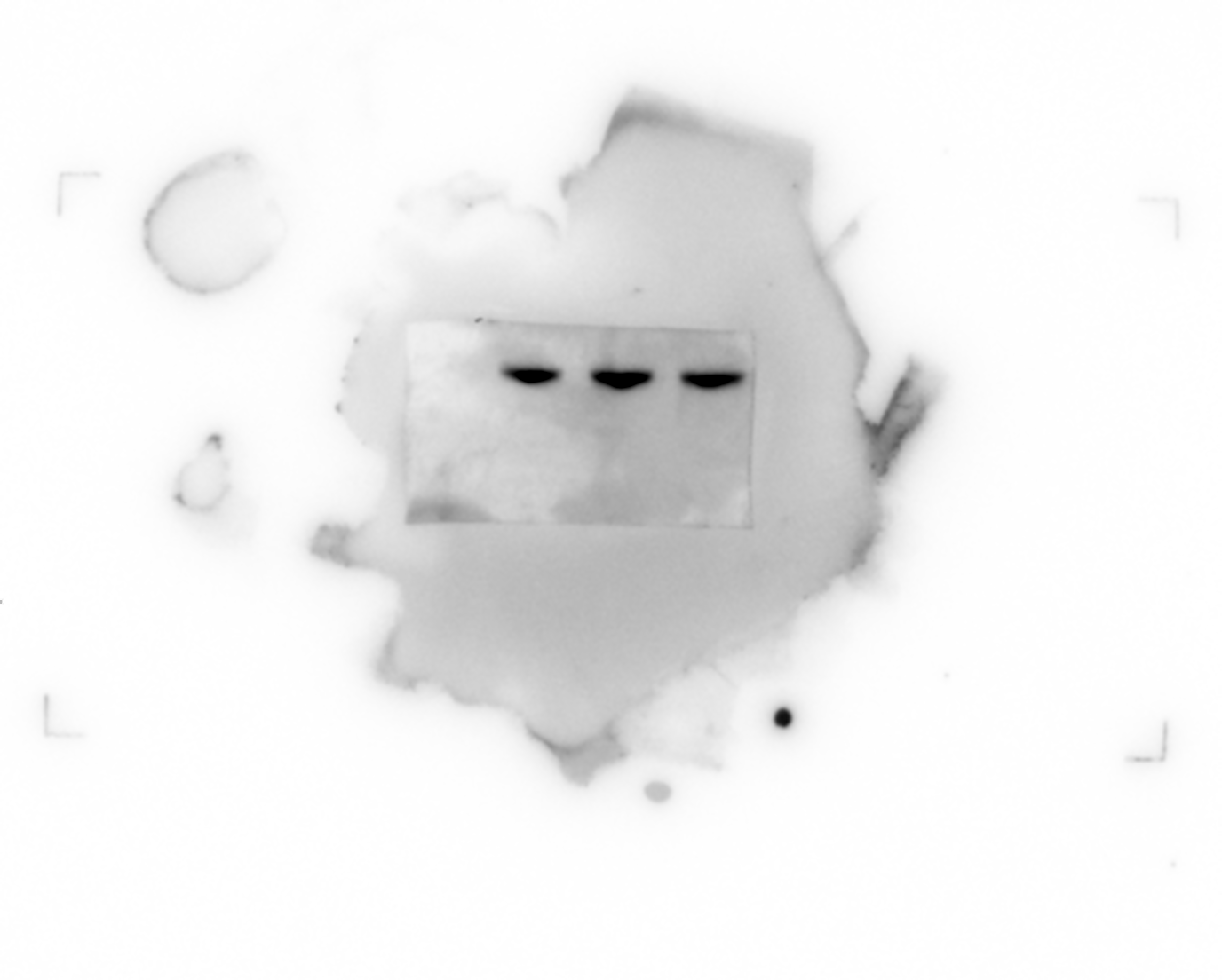


CDK5

###
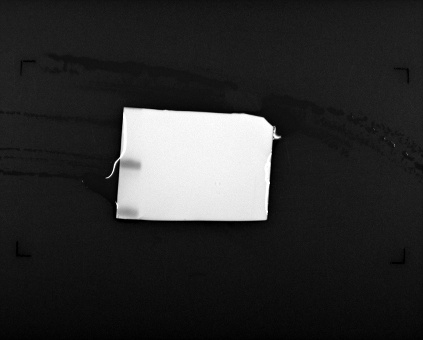

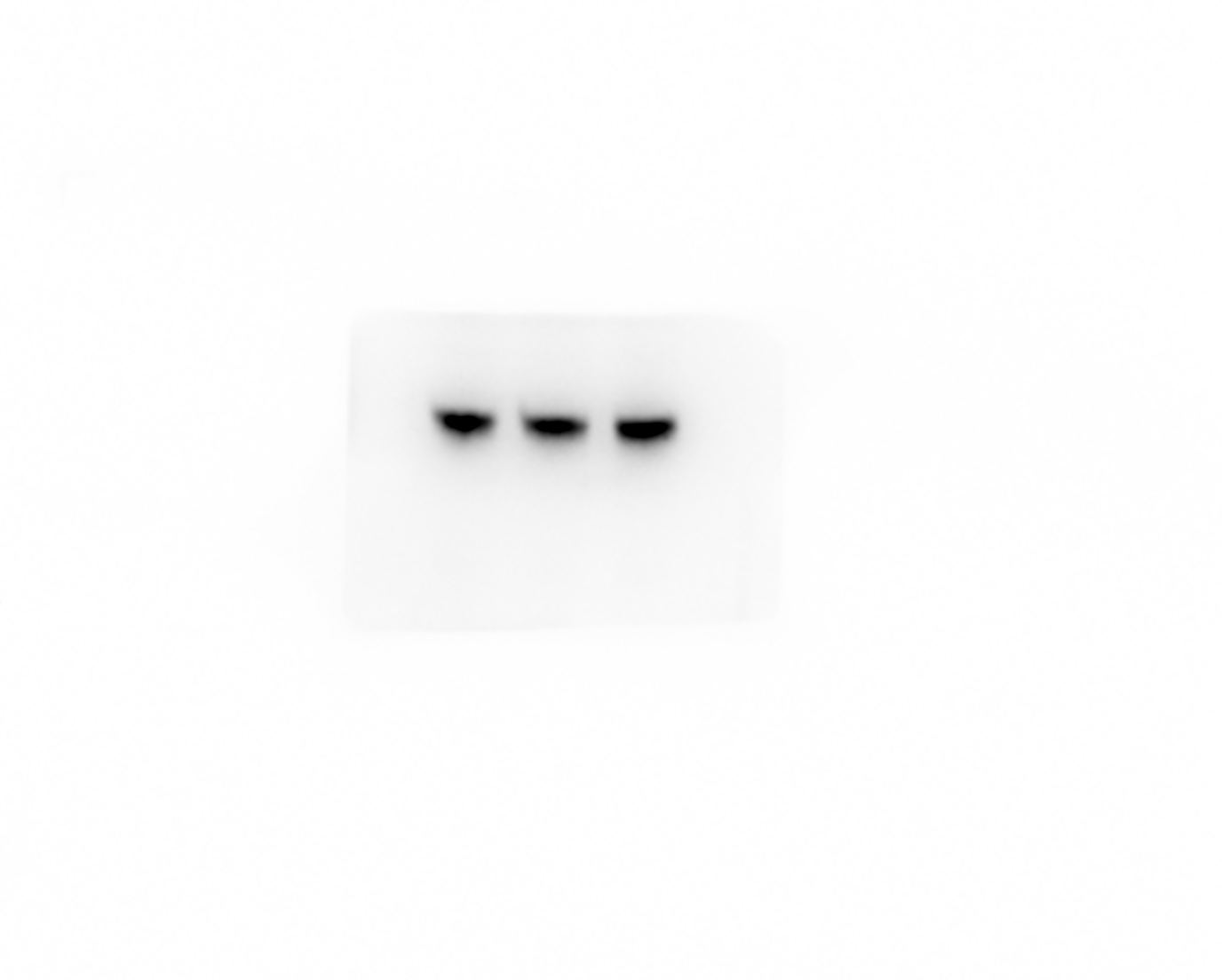


EPHA4

###
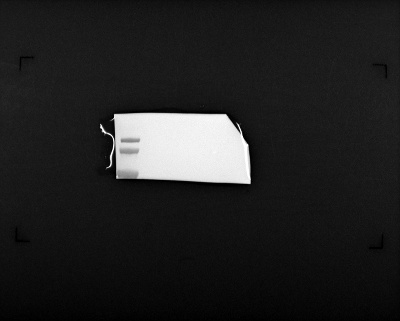

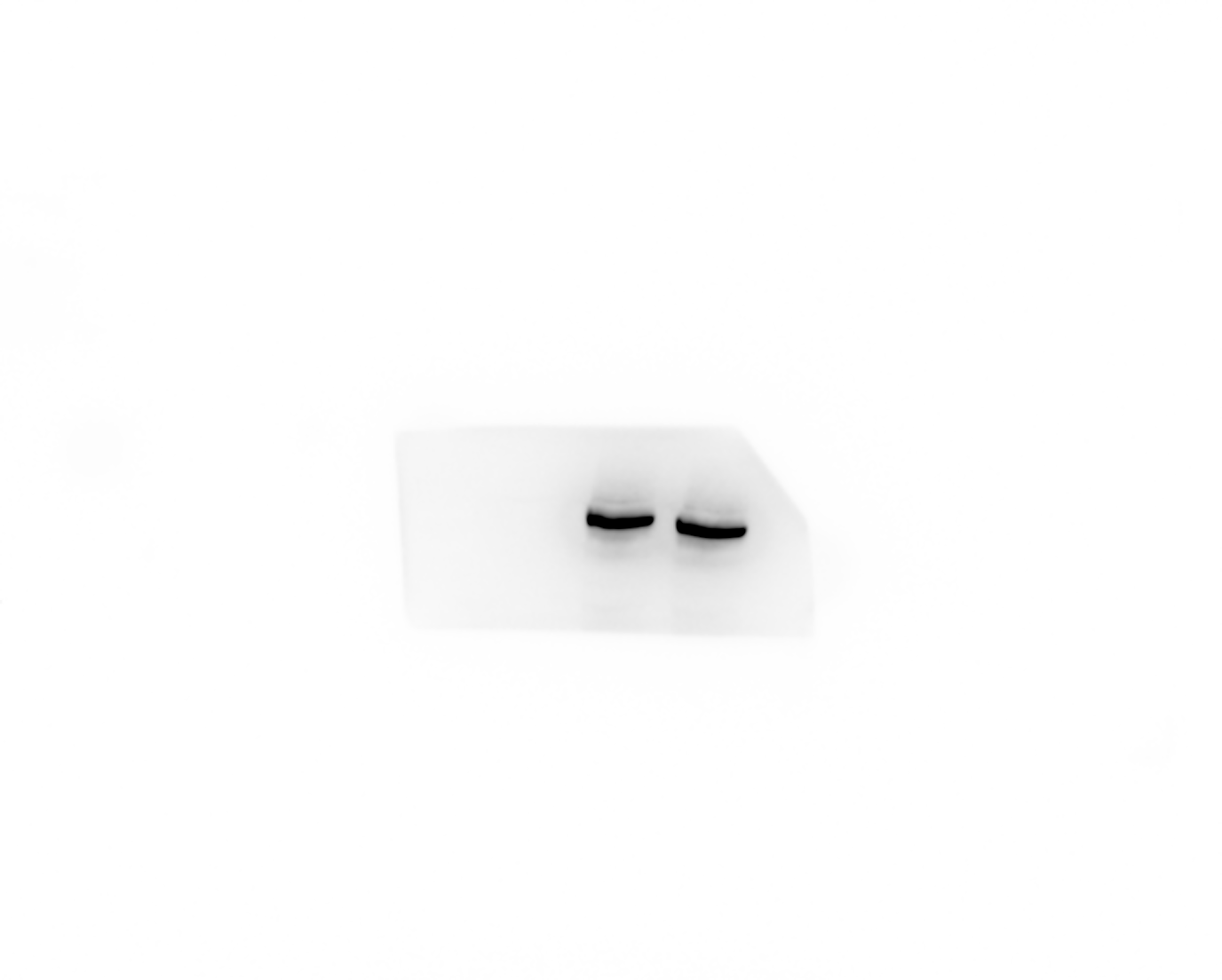


β-actin

###
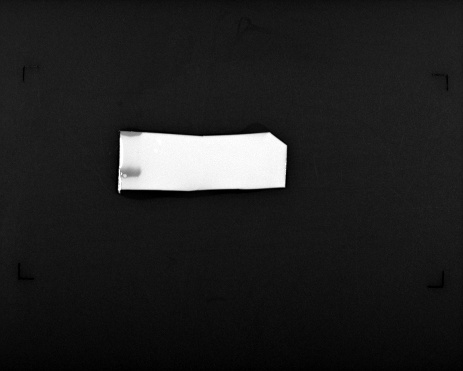

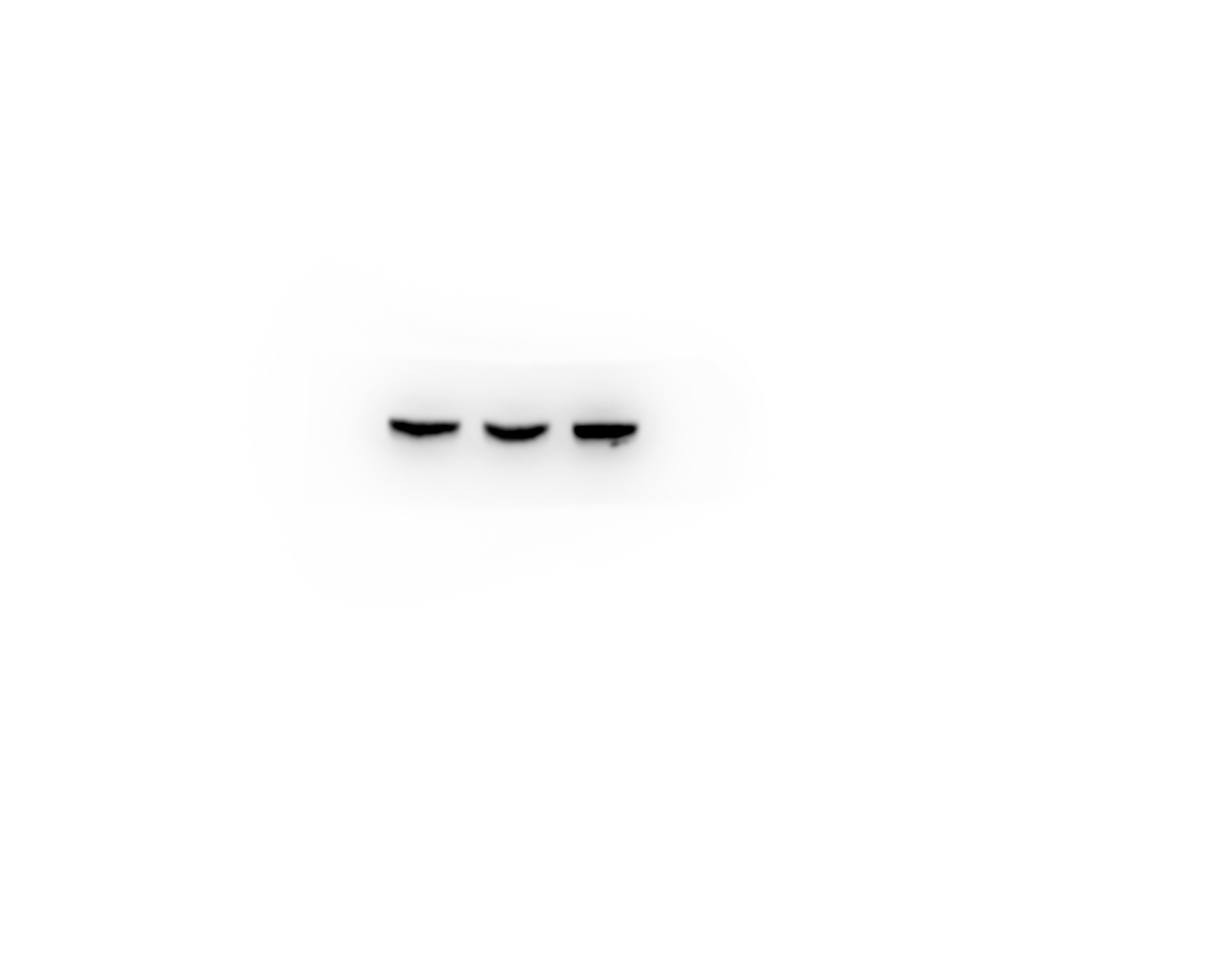

Supplement: Figure 1—source data 1. [file elife-95324-fig1-data1.docx]
