# Supplementary material for: EPHA4 signaling dysregulation links abnormal locomotion and the development of idiopathic scoliosis
Source: eLife. 2025 Jul 15;13:RP95324. doi: 10.7554/eLife.95324 (PMC12263152; doi:10.7554/eLife.95324)
Supplement: Supplementary file 1. [file elife-95324-supp1.docx]

### **Supplementary file 1. Summary of the 14 studies and their corresponding SNPs included in the candidate genes mapping.**

| **Citation** | **SNPs** |
| --- | --- |
| Fan et al., J Hum. Genet. 2012 | rs11190870 (p= 9.1 × 10^-10) |
| Zhu et al., Hum. Mol. Genet. 2017 | rs7593846 (p= 1.19 × 10^-13)  rs7633294 (p= 1.85 × 10^-12)  rs6570507 (p= 1.14 × 10^-11)  rs6047663 (p= 1.61 × 10^-15) |
| Zhu et al., Nat. Commun. 2015 | rs678741 (p= 9.68 × 10^-37)  rs4940576 (p= 2.22 × 10^-12)  rs13398147 (p= 7.59 × 10^-13)  rs241215 (p= 2.95 × 10^-9) |
| Takahashi et al., Nat. Genet. 2011 | rs11190870 (p= 1.24 × 10^-19)  rs625039 (p= 8.13 × 10^-15)  rs11598564 (p= 5.98 × 10^-14) |
| Sharma et al., Hum. Mol. Genet. 2011 | rs1400180 (p= 6.35 × 10^-8) |
| Kou et al., Nat. Genet. 2013 | rs6570507 (p= 1.27 × 10^-14) |
| Xu et al., Spine Deform. 2018 | rs169311 (p= 2.10 × 10^-8) |
| Grauers et al., Spine J. 2015 | rs11190870 (p= 7.0 × 10^-18) |
| Ogura et al., Am. J of Hum. Genet. 2015 | rs3904778 (p= 2.46 × 10^-13) |
| Khanshour et al., Hum. Mol. Genet. 2018 | rs4513093 (p= 1.71 × 10^-15)  rs1455114 (p= 2.99 × 10^-8)  rs687621 (p= 7.29 × 10^-10)  rs10756785 (p= 7.00 × 10^-10) |
| Sharma et al., Nat. Commun. 2015 | rs6137473 (p= 3.12 × 10^-8) |
| Miyake et al., PLoS One 2013 | rs11190870 (p=2.80 × 10^-18)  rs625039 (p= 1.28 × 10^-15)  rs12946942 (p= 6.43 × 10^-12)  rs11598564 (p= 9.77 × 10^-12)  rs6570507 (p= 3.78 × 10^-8)  rs9496346 (p= 1.00 × 10^-8) |
| Chettier et al., PLoS One 2015 | rs11190878 (p= 4.18 × 10^-9) |
| Kou et al., Nat. Commun. 2019 | rs9389985 (p= 3.51 × 10^-20)  rs7028900 (p= 2.19 × 10^-17)  rs144131194 (p= 1.35 × 10^-11)  rs6047716 (p= 1.45 × 10^-11)  rs141903557 (p= 9.78 × 10^-11)  rs11205303 (p= 1.62 × 10^-10)  rs12029076 (p= 2.17 × 10^-10)  rs1978060 (p= 3.26 × 10^-10)  rs2467146 (p= 5.96 × 10^-10)  rs11787412 (p= 1.32 × 10^-9)  rs188915802 (p= 1.94 × 10^-9)  rs658839 (p= 3.15 × 10^-9)  rs2194285 (p= 8.69 × 10^-9)  rs160335 (p= 9.10 × 10^-9)  rs482012 (p= 2.30 × 10^-8)  rs11341092 (p= 2.92 × 10^-8)  rs17011903 (p= 3.56 × 10^-8)  rs397948882 (p= 3.66 × 10^-8)  rs12149832 (p= 4.40 × 10^-8) |
