# Supplementary material for: EPHA4 signaling dysregulation links abnormal locomotion and the development of idiopathic scoliosis
Source: eLife. 2025 Jul 15;13:RP95324. doi: 10.7554/eLife.95324 (PMC12263152; doi:10.7554/eLife.95324)
Supplement: Supplementary file 2. [file elife-95324-supp2.docx]

### **Supplementary file 2. Summarized results of burden analysis.**

| **Rank** | **Gene Symbol** | ***P* value** | **Variants in Cases (N = 411)** | **Variants in Controls (N = 3,800)** | **OR (95%CI)** |
| --- | --- | --- | --- | --- | --- |
| 1 | *EPHA4* | 0.045533 | 3 | 17 | 4.09 (0.946-17.6) |
| 2 | *ARVCF* | 0.067987 | 6 | 24 | 3.05 (0.807-11.6) |
| 3 | *TXNRD2* | 0.092043 | 3 | 11 | 3.60 (0.729-17.8) |
| 4 | *TRIM17* | 0.115033 | 3 | 11 | 3.53 (0.795-15.7) |
| 5 | *SLC25A1* | 0.119166 | 3 | 9 | 3.39 (0.817-14.1) |
| 6 | *MICALL2* | 0.199131 | 0 | 24 | 0.30 (0.0176-4.94) |
| 7 | *MTMR11* | 0.249127 | 5 | 35 | 2.05 (0.663-6.36) |
| 8 | *MMP2* | 0.252208 | 4 | 17 | 3.03 (0.613-15.0) |
| 9 | *BCKDHB* | 0.293142 | 1 | 16 | 0.643 (0.0469-8.83) |
| 10 | *TTK* | 0.293162 | 0 | 17 | 0.583 (0.0334-10.2) |
| 11 | *SLC2A6* | 0.316088 | 1 | 21 | 0.675 (0.049-9.3) |
| 12 | *SOX6* | 0.317369 | 2 | 25 | 0.822 (0.0949-7.11) |
| 13 | *WNT3A* | 0.329116 | 1 | 11 | 0.814 (0.058-11.4) |
| 14 | *SDR42E1* | 0.33538 | 0 | 11 | 0.631 (0.0359-11.1) |
| 15 | *GPR126* | 0.33628 | 0 | 12 | 0.699 (0.0393-12.4) |
| 16 | *PIGN* | 0.338169 | 1 | 22 | 0.663 (0.0869-5.06) |
| 17 | *POLL* | 0.348494 | 0 | 11 | 0.668 (0.0378-11.8) |
| 18 | *PAX1* | 0.350574 | 0 | 19 | 0.649 (0.0368-11.4) |
| 19 | *INSC* | 0.363501 | 0 | 13 | 0.744 (0.0416-13.3) |
| 20 | *IBA57* | 0.388644 | 0 | 8 | 0.744 (0.0416-13.3) |

The p-values (Fisher's Exact Test) of 20 top ranked gene were calculated on the basis of distribution differences of rare variant between the control group and the PUMCH IS cohort. Abbreviations: OR (odds ratio); CI (confidence interval).
