# Supplementary material for: EPHA4 signaling dysregulation links abnormal locomotion and the development of idiopathic scoliosis
Source: eLife. 2025 Jul 15;13:RP95324. doi: 10.7554/eLife.95324 (PMC12263152; doi:10.7554/eLife.95324)
Supplement: Supplementary file 4. [file elife-95324-supp4.docx]

### **Supplementary file 4. Search strategies for each database.**

| **Search Number** | **Key Words** | **Results** |
| --- | --- | --- |
| **MEDLINE (via Pubmed.gov)** | | |
| 1# | Idiopathic scoliosis, GWAS/ | 69 |
| 2# | Idiopathic scoliosis, SNP/ | 56 |
| 3# | Idiopathic scoliosis, single nucleotide polymorphism/ | 149 |
| 4# | Idiopathic scoliosis, variant/ | 129 |
| **Web of Science (via Clarivate Analytics)** | | |
| 1# | Idiopathic scoliosis, GWAS/ | 42 |
| 2# | Idiopathic scoliosis, SNP/ | 71 |
| 3# | Idiopathic scoliosis, single nucleotide polymorphism/ | 198 |
| 4# | Idiopathic scoliosis, variant/ | 227 |
