# Supplementary material for: EPHA4 signaling dysregulation links abnormal locomotion and the development of idiopathic scoliosis
Source: eLife. 2025 Jul 15;13:RP95324. doi: 10.7554/eLife.95324 (PMC12263152; doi:10.7554/eLife.95324)
Supplement: Supplementary file 5. [file elife-95324-supp5.docx]

### **Supplementary file 5. Inclusion and exclusion criteria of literature review.**

|  | **Inclusion Criteria** | **Exclusion Criteria** |
| --- | --- | --- |
| Patient Recruitment | Idiopathic scoliosis  Adolescent idiopathic scoliosis  Early-onset idiopathic scoliosis | Congenital scoliosis  Neuromuscular scoliosis  Syndromic scoliosis  Secondary scoliosis  Degenerative scoliosis |
| Study Type | Genome Wide Association Study (GWAS)  Meta-analysis of GWAS | Linkage study  Candidate gene study  Exome sequencing  Whole genome sequencing |
| Study Design | Case-control | - |
