# Supplementary material for: EPHA4 signaling dysregulation links abnormal locomotion and the development of idiopathic scoliosis
Source: eLife. 2025 Jul 15;13:RP95324. doi: 10.7554/eLife.95324 (PMC12263152; doi:10.7554/eLife.95324)
Supplement: Supplementary file 6. [file elife-95324-supp6.docx]

### **Supplementary file 6. Sequencing information of PUMCH IS cohort.**

| **Characteristics** | **Cases** | |  | **Controls** | |
| --- | --- | --- | --- | --- | --- |
|  | **Trio** | **Singleton** |  | **Trio** | **Singleton** |
| Exome sequencing | 41 | 113 |  | 2,021 | 224 |
| Genome sequencing | 116 | 148 |  | 483 | 1,072 |
| Sum | 157 | 261 |  | 2,504 | 1,296 |
| Passing QC | 155 | 256 |  | 2,504 | 1,296 |

Abbreviations: QC (quality control); IS (idiopathic scoliosis).
