# Supplementary material for: EPHA4 signaling dysregulation links abnormal locomotion and the development of idiopathic scoliosis
Source: eLife. 2025 Jul 15;13:RP95324. doi: 10.7554/eLife.95324 (PMC12263152; doi:10.7554/eLife.95324)
Supplement: Supplementary file 7. [file elife-95324-supp7.docx]

### **Supplementary file 7. Sequences information of sgRNA and primers for zebrafish study.**

| sgRNA sequences used to generate zebrafish *epha4a* and *epha4b* mutants | |
| --- | --- |
| *epha4a* sgRNA-1 | AAGATTAAAGGTCTCCTTGC |
| *epha4a* sgRNA-2 | GTATCGCTCTTTGTCATTGT |
| *epha4a* sgRNA-3 | GAAGCTTTCATCAGCCGCAA |
| *epha4a* sgRNA-4 | CCGTGTTCAGCTTCATGATG |
| *epha4b* sgRNA-1 | GCCAAGTTCAACACCG-CCAG |
| *epha4b* sgRNA-2 | AGTCACCGTATCGGGAAACT |
| Primers for mutant genotype | |
| *epha4a*-forward | GAGCTCAGCGGGTCTACATC |
| *epha4a*-reverse | GCGTACCTCCACTAGCGATG |
| *epha4b*-forward | GGTGTCTCTGAGGGTCTTTT |
| *epha4b*-reverse | CCTGACAGTAGTCCTTATTCTCCTC |
| Morpholinos used to knock down the expression of *epha4a* and *efnb3b* | |
| *epha4a* MO | AACACAAGCGCAGCCATTGGTGTC |
| *efnb3b* MO1 | GTCTATTTACTCCCATCAAAGCCGT |
| *efnb3b* MO2 | GAAATCCCGAATTCCGTCTATTTAC |
| Primers used for in situ hybridization analysis | |
| *epha4a* probe forward | AGAATACGCCAATCAGGACG |
| *epha4a* probe reverse | CCTTCATGTTCTTCATAGCC |
| *epha4b* probe forward | TTTGGTGGAGGTTAGAGGGT |
| *epha4b* probe reverse | GTTGTTTAGGGGTTGGGCTC |
| *rfng* probe forward | GGTGCTGCTTATTCCCTCCTT |
| *rfng* probe reverse | GCGCTAAACCTCGACTGATGC |
